# Breed influences on *in vitro* development of abattoir-derived bovine oocytes

**DOI:** 10.1186/1751-0147-54-36

**Published:** 2012-06-10

**Authors:** M Celina Abraham, Hans Gustafsson, Alejandro Ruete, Ylva CB Brandt

**Affiliations:** 1 Department of Clinical Sciences, Division of Reproduction, Swedish University of Agricultural Sciences (SLU) Uppsala, Stockholm, Sweden; 2 Swedish Dairy Association, Stockholm, Sweden; 3 Department of Ecology, Swedish University of Agricultural Sciences (SLU) Uppsala, Stockholm, Sweden

**Keywords:** Cow, Heifer, IVF, IVM, IVP, Fertility, Blastocyst

## Abstract

**Background:**

There is a discrepancy in the reproductive performance between different cattle breeds. Using abattoir-derived ovaries and data base information we studied the effects of breed on *in vitro* fertilization and early embryo development.

**Methods:**

The *in vitro* developmental competence of oocytes from cattle (n = 202) of Swedish Red (SR), Swedish Holstein (SH) and mixed beef breeds was compared, retrospectively tracing donors of abattoir-derived ovaries using a combination of the national animal databases and abattoir information. Age was significantly lower and carcass conformation score was higher in the beef breeds than in the dairy breeds.

Cumulus oocyte complexes (n = 1351) were aspirated from abattoir-derived ovaries from animals of known breed (visual inspection confirmed through databases), age (databases), and abattoir information. Oocytes were matured, fertilized (frozen semen from two dairy bulls) and cultured according to conventional protocols. On day 8, blastocysts were graded and the number of nuclei determined.

**Results:**

Cleavage rate was not different between the breeds but was significantly different between bulls. The percentage of blastocysts on day 8 was significantly higher when the oocyte donor’s breed was beef or SR than SH. There was no significant difference in blastocyst grades or stages between the breeds, but the number of nuclei in day 8 blastocysts was significantly lower in SH compared to the beef.

**Conclusions:**

The use of abattoir-derived ovaries from animals whose background is traceable can be a valuable tool for research. Using this approach in the present study, oocyte donor breed was seen to affect early embryo development during *in vitro* embryo production, which may be a contributing factor to the declining fertility in some dairy breeds seen today.

## Background

Reproductive performance is dependent on many factors, one of which is oocyte quality and thereby the ability of the oocyte to be fertilized and develop normally [1]. It is known that the quality of the oocyte is the main factor affecting blastocyst yields in *in vitro* production systems [2]. Moreover the developmental competence and the morphology of the embryo are related to pregnancy rate following transfer and therefore the chance for a live calf [3]. The use of the *in vitro* approach to oocyte developmental competence could eliminate the effects of some cow management factors e.g. estrus detection, timing of artificial insemination (AI) relative to ovulation and AI-procedures.

Obtaining sufficient numbers of oocytes from animals with known identity and background can present a problem for scientific studies since it is seldom possible to trace the individual animal through the production line in the abattoir to the point where ovaries can be collected and instantly gain information on the animal background. In Sweden, the national recording system (Swedish Official Milk Recording Scheme (Swedish Dairy Association) and the Swedish Official Beef Recording Scheme) [4] can be used to trace background data, such as age, breed, milk production (dairy breeds), number of calvings, reason for slaughter, fertility, prior diseases, treatments and farm of origin of the individual animals. In this study, breed was selected as the major parameter to study since it would be possible to make a preliminary breed classification in the slaughterhouse based on the gross inspection of the slaughtered animal when the ovaries were collected and thereby make a breed-wise pooling of the collected oocytes.

There is a discrepancy in the reproductive performance between different cattle breeds indicating better fertility in beef breeds compared to dairy breeds [5,6]. In addition there has been a pronounced negative fertility trend in high-yielding dairy cows during recent decades [7,8]. However, differences in fertility have even been observed between dairy breeds, with better fertility in the SR dairy breed compared to e.g. the American Holstein breed [9,10]. It is not known if these differences are caused by inherent factors affecting the reproductive potential or are a result of other factors, such as changes in management coupled to increasing herd size. To our knowledge, there are few studies comparing the oocyte quality in different cattle breeds as a potential factor for fertility.

This study aimed to test the possibility of using abattoir-derived ovaries for *in vitro* embryo production where the identity of the donor animal was known and to use this information to study the effects of breed on *in vitro* fertilization and early embryo development. This opportunity could provide the possibility of comparing different animal factors retrospectively through the unique use of combined animal databases and abattoir information.

Part of this data was presented at the 36th Annual Conference of the IETS/23rd Annual Meeting of the SBTE, 2010 [11].

## Methods

### Experimental design

At the slaughterhouse each carcass was visually classified as dairy or beef breed followed by the collection of ovaries in pairs (between 8:00 - 10:00 am) with the corresponding donors IDs. After transportation to laboratory the cumulus oocyte complexes (COC) (n total = 1351: SR = 448; SH = 290; beef = 613) were aspirated and pooled within dairy and beef breeds for subsequent IVM/IVF/IVC. The experiment was run in 18 batches. The oocytes were fertilized with frozen semen from two different AI bulls with proven field fertility, one of the SR breed and one of the SH breed. The bulls were used alternately for a total number of 18 batches (nine batches per bull and the same bull in even or uneven numbered batches). Embryo development was followed and cleavage rates at 44 hours post insemination (hpi) and blastocyst rates on days 7 and 8 were recorded. On day 8 the morphological quality of embryo was assessed and the total cell counts of blastocysts were calculated. All *in vitro* procedures (IVP) were consistent throughout the experiment. Weeks after the IVPs data concerning background information of oocyte donors were collected from the data bases based on the IDs. Due to errors in visual breed allocation at the slaughterhouse some IVP batches were removed from the data before statistical analyses (see below). All media and constituents were obtained from Sigma-Aldrich (Stockholm, Sweden) unless otherwise stated.

### Animals, abattoir information and database information

A preliminary breed classification was done in the abattoir by visual inspection. Carcass conformation and fatness scores were subjectively evaluated by trained graders in accordance with the European classification scheme S/EUROP system at the commercial slaughterhouse. The carcasses were weighed warm shortly after evaluation.

Almost all animals were found in the databases (209 out of 211) and the visual classification of the breed was mostly correct (202 out of 209). These 202 animals were from 67 different farms. The final breed distribution was as follows: 71 animals of the SR breed, 26 animals of the SH breed and 105 animals of various beef breeds (Charolais: 24, Aberdeen Angus: 11, Hereford: 7, Blonde d’ Aquitaine: 5 and crossbred beef breeds: 58).

### Retrieval of ovaries and cumulus oocyte complexes (COCs)

The ovaries were clamped together pair-wise with an ID-tag, pooled in 0.9% NaCl solution at 38 C and processed in the laboratory within 3.5 - 4 h of collection. The breed of each animal was estimated through a visual inspection at the slaughterhouse. Aspirates were made from follicles with diameters ranging between 3 - 8 mm using a 5 ml syringe and 18-gauge hypodermic needle and were collected into search medium (HEPES-buffered Medium 199 with 0.2% w/v BSA fraction V and 50 IU/ml penicillin and 50 μg/ml streptomycin), pooling those from the same breeds. From the search medium, only COCs of excellent or good quality were selected for maturation (oocytes category 1 - 3, according to Goodhand et al. [12]). Each batch was divided in two to three pools depending on how many breeds were retrieved at the abattoir, giving a total of 13 pools of SR, 12 pools of SH and 18 pools of beef. The pool sizes were between 20-50 COCs (on average: Beef: 34, SH: 28 and SR: 29 COCs).

### *In vitro* maturation, fertilization and culture

The maturation media consisted of TCM 199 with additions of L-glutamine (0.1 mg/ml), LH (10 μg/ml), FSH (10 μg/ml), 0.4% w/v BSA fraction V, 50 IU/ml penicillin and 50 μg/ml streptomycin. The maturing COCs were incubated in groups of 20 - 50 in 500 μl maturation media in an atmosphere of 5% CO_2_ in humidified air at 38.5 ° C for 24 h. After maturation, the COCs were removed from maturation media to wash media consisting of modified Tyrode’s albumin lactate pyruvate (mTALP, containing 0.3% w/v fraction V BSA) [13]. They were pipetted to mechanically remove all but the closest 3 - 5 layers of cumulus cells surrounding each oocyte. The oocytes were then transferred to wells with 500 μl fertilization media (mTALP containing 0.6% w/v fatty acid free BSA, 3 μg/ml heparin, 3 μg/ml penicillamine, 3 μg/ml epinephrine and 1.1 μg/ml hypotaurine). The spermatozoa were thawed and prepared by the swim-up procedure in capacitation media (mTALP without CaCl_2_, containing 1.25 mg/ml glucose and 0.6% w/v fraction V BSA) for 45 minutes at 38.5 C in a 5% CO_2_ incubator. After swim-up, the spermatozoa were washed and concentrated by centrifugation (300 × g) for 8 minutes and finally added to the oocytes at a concentration of 1 x 10^6^ spermatozoa/ml. The oocytes were incubated with the spermatozoa in an atmosphere of 5% CO_2_ in humidified air at 38.5 ° C for 22 h. Culture media consisted of synthetic oviduct fluid (SOF) [14] without antibiotics. At 22 hpi the oocytes were denuded by pipetting and cultured in groups of 20 –50 in 500 μl SOF media in a humified atmosphere of 5% CO_2_, 5% O_2_ and 90% N_2_ at 38.5 ° C and wells were covered with mineral oil. At 44 hpi, the numbers of uncleaved and cleaved embryos were noted as well as the number of embryos beyond the 2-cell stage. The number of blastocysts developed by day 7 and day 8 were recorded (168 hpi 7 and 192 hpi). On day 8, all blastocysts were graded according to Lindner and Wright [3]. The cleavage rates and blastocyst rates were calculated from the number of oocytes.

### Fixation and staining

On day 8, the embryos were fixed with paraformaldehyde (diluted to 2% with Dulbeccos PBS with 1% PVA) overnight at 4 °C. They were washed 2 - 3 times in PBS with PVA. After that, they were incubated for 15 minutes with Hoechst 33342 (2.5 μg/ml) and washed 3 times with PBS with PVA. The embryos were mounted with a mix of vaseline and paraffin on glass slides and the number of nuclei was recorded twice for every blastocyst by two independent persons using epifluorescence microscopy (Leitz GmbH Dialux 20, Wetzlar, Germany).

### Statistical analysis

For abattoir and database data, the effect of breed on individual cow data (age, carcass conformation score, fatness score and body weight) was fitted with linear regression models for continuous data, and with ordinal logistic regression models for ordered categorical data (carcass conformation score and fatness score). Models were fitted in R v2.11 [15] with the add-on library Zelig [16]. The effect of the independent variable oocyte donor’s breed on the different dependent variables -i.e. cleavage rate, number of cleaved beyond the 2-cell stage, number of blastocysts at day 7 and at day 8, and quality grade was tested using Logistic regression models with the intercept as random effect and batch as grouping variable. The effect of the origin of the semen was also tested. A Poisson regression model was fitted to test for the effect of breed on the number of nuclei in blastocysts at day 8, with the intercept as random effect and batch as grouping variable. The effect of the variable oocyte donor’s breed was assessed based on the improvement of the fit of the model (likelihood-ratio test, last two columns in Table 1; [17]). The models were fitted in R with the add-on library lme4 [18,19].

**Table 1:** **Significance of the oocyte donor’s breed on the number (mean ± SD (n)) of oocytes developed into different maturation stages, after *in vitro *production**

**Variable**	Beef*	**SH**	**SR**	**LRT**	**P**
**Cleavage rate % (n)**	73.9 ± 0.03 (613)^a^	72.5 ± 0.02 (290)^a^	71.9 ± 0.03 (448)^a^	Chisq = 3.77 (df = 2)	0.150
**Cleaved beyond 2-cell stage at 44 hpi % (n)**	44.9 ± 10.6 (613)^a^	44.2 ± 6.95 (290)^b^	52.1 ± 6.98 (448)^ab^	Chisq = 1.008 (df = 2)	0.60
**Blastocysts developed by day 7 (168 hpi) % (n)**	8.8 ± 3.33 (613)^a^	3.2 ± 3.19 (290)^a^	5.9 ± 5.8 (448)^a^	Chisq = 3.71 (df = 2)	0.156
**Blastocysts developed by day 8 (192 hpi) % (n)**	14 ± 7.6 (613)^a^	7.4 ± 4.5 (290)^b^	15.3 ± 0.6 (448)^a^	Chisq = 8.13 (df = 2)	0.0156
**Number of nuclei in day 8 blastocysts**	101.4 ± 6.9^a^	79.2 ± 8.7b	98.9 ± 7.7^ab^	Chisq = 23.02 (df = 2)	<0.001
**Blastocyst grades day 8 (1 – 4 where 1 = best grade)**	2.1 ± 0.2^a^	2.4 ± 0.1^a^	2.4 ± 0.2^a^	LR = 4.19 (df = 2)	0.123

Cleavage and blastocyst ratios are calculated from number of oocytes inseminated with dairy bull semen. In each row, different letters after data indicates statistically significant differences between oocyte donor’s breeds. LRT is the likelihood-ratio test (statistical values), and P are p-values that represent the significance of the effect of the independent variable oocyte donor’s breed on the dependent variable. * The beef breed oocyte donors deviated in age and carcass conformation compared to the dairy breeds (p < 0.05).

## Results

The age was significantly lower in the beef breeds (mean age: 3.8 years) than in SH (mean age: 5.0 years) and SR (mean age: 5.2 years) (p = 0.005). The mean carcass weight was similar between all breeds (Beef: 310 kg, SH: 318 kg, SR: 300 kg) (p = 0.37). Carcass conformation was scored significantly higher in the beef breeds than in the dairy breeds (p = 0.003) but there was no difference in fatness score between the groups (p = 0.930). The mean number of oocytes retrieved/ ovary did not differ between breeds (Beef: 3.33 ± 1.03, SH: 3.53 ± 1.20, SR: 3.74 ± 1.19).

Cleavage rate was not different between the breeds (Table 1). There was a significant difference in cleavage rate between the two bulls used (Chisq = 3.912, df = 1, p = 0.048). All of the variability in the percentage of embryos developed beyond the 2-cell stage could not be explained by the variable oocyte donor’s breed. However, the percentage of embryos developed beyond the 2-cell stage at 44 hpi was significantly greater for SH oocyte donor than for the beef oocyte donors’ breeds (z = 2.230, p = 0.025). The percentage of blastocysts developed by day 8 was significantly higher in the beef oocyte donors’ breed group and SR compared to SH (Table 1). There was no significant difference in blastocyst grades among the oocyte donors’breeds, but the number of nuclei 9 in blastocysts on day 8 was significantly lower in SH than in beef breeds (Table 1). Including the variable blastocysts stage improved the explanatory power of the model for number of nuclei in day 8 blastocysts (Chisq = 794.630, df = 4, *p* = >0.001); but blastocysts stage by itself was not significantly different between the breeds.

## Discussion

In this study the *in vitro* developmental competence of oocytes was investigated between different cattle breeds. One difference between the beef breeds and the dairy breeds among the parameters measured in this study was the age (where beef breeds generally were younger at the time of slaughter). Donor age has been suspected to play a role in developmental competence of bovine oocytes, but whether the young cows or heifers or the older cows have a better oocyte quality remains unclear [20,21]. Body condition is dependent on both carcass conformation classification and fatness score. In our study the conformation classification of the carcasses was significantly higher in the beef breeds compared to the dairy breeds. This is not surprising but could be connected to the tendency towards the better oocyte quality seen in the beef group since body condition can affect the quality of the follicles [22]. When comparing beef and dairy cattle, husbandry-driven stress could be one important cause for the reduced fertility in dairy cattle, causing repeat breeding and early embryonic death [23,24]. Beef cattle are not bred for high milk production and avoid most of the husbandry and milk production related stressors and should therefore have retained a better fertility.

The semen donor also affects the ability of the embryo to develop and survive throughout pregnancy [8]. In our study two dairy bulls were used and these gave significantly 10 different cleavage rates regardless of oocyte donor breed. This finding, interestingly, was consistent with field data for the respective bull, since the field fertility of the bull with significantly lower cleavage rates in IVF was 5% lower than average field fertility of all the the Swedish AI bulls at the time for the study while the other bull had an average field fertility based on non return rate data from the Swedish Dairy Association. This data was in agreement with Al Naib et al. [25] who found differences in cleavage rate but not in blastocysts rate for different individual bulls.

Heterosis is defined as increased vigour of growth, survival, and fertility of hybrids, the adult generation as a result of crossbreeding (F1 generation). Results from different studies with Holstein crossed with e.g. Scandinavian red breeds have shown that the crosses had better fertility (shorter days open period) than purebred Holstein [26]. In this experiment the oocytes were not always inseminated with homozygous semen. However we are not aware of any reports showing differences of homozygous or heterozygous inseminations of oocytes *in vitro*.

By using abattoir-collected ovaries a large number of oocytes from different individuals were available. Although it was not possible to follow oocytes from each individual animal through the process of *in vitro* maturation, fertilization or culture, an approach that would be ideal, oocytes were instead pooled according to breed. This was done because oocytes cultured in groups have been observed to be developmentally more competent than oocytes kept in individual culture [27,28].

The reporting of correct data into the databases is difficult to verify but in a study of these particular databases where the farmer and veterinarian reports have been compared, the completeness was high [29]. Also, there is a delay in reporting AI by some farmers performing inseminations which may make some fertility measures incorrect [30]. As soon as data are reported, it is available in the database within 24 h. In the future, hopefully faster registration of data into the database system can provide us and other research groups with reliable information regarding individual animals, other than breed or traits that can be recorded at the time of slaughter and ovary retrieval.

In the study reported here, the beef and SR oocyte donors’ breeds displayed a higher blastocyst percentage, and the beef also had higher number of nuclei in the blastocysts on day 8 of *in vitro* culture indicating a better oocytes quality than SH. This is consistent with field data showing a 3 - 5% better conception rate after AI in SR compared to SH [8]. Our results indicate that there is a difference in oocyte quality between the two dairy breeds, which might be due to other reasons than herd factors. However, the difference seen may have been due to SH being more sensitive to the *in vitro* production system than SR. The reason for the better fertility seen in SR is most likely that it has been consistently bred for good fertility traits for several decades. Also, SR has a slightly lower milk yield than SH which may cause less metabolic stress [31].

This study did not indicate any differences in total cleavage rates between the oocyte donors’ breeds investigated, but the SH group surprisingly displayed a higher number of embryos cleaved beyond the 2-cell stage at 44 hpi than the beef group. In general, early development is believed to be an indicator of good developmental ability in bovine *in vitro* embryo production [32-34], a concept that was contradicted by the final blastocyst rate in this study since the SH group had the lowest blastocyst rate on day 8. It is possible that the SH group suffered from an extensive 8 - 16 cell block but since only blastocysts were assessed after culture, the main point of developmental arrest is not clear. There is also a study in cattle suggesting that embryos developing either too slow or too fast have less potential to reach the blastocyst stage *in vitro*, possibly due to a higher incidence of chromosomal abnormalities [35].

In our study the beef oocyte donors’ breeds tended to be superior or equal to SR. However, most of the significant differences were only seen between the beef and the least successful dairy breed (SH).

## Conclusions

The use of abattoir-derived ovaries from animals whose background can be traced, is a valuable tool for research that should be further explored when proceeding with for example *in vitro* production of oocytes. In this study, the breed of origin of the cattle oocytes affected early embryo development during *in vitro* embryo production. This suggests oocyte quality is an important factor behind the difference in fertility we can see among cattle breeds.

## Competing interests

The authors declare that they have no competing interests.

## Authors’ contributions

MCA and YCB designed the study and did the laboratory work. HG participated in the design and coordination of the study. AR performed the statistical analysis. All authors read, discussed the draft, improved the writing and approved the final manuscript.

## Authors’ information

Department of Clinical Sciences, Division of Reproduction, Swedish University of Agricultural Sciences (SLU) Uppsala, Sweden ^2^ Swedish Dairy Association, Stockholm, Sweden. ^3^Department of Ecology, Swedish University of Agricultural Sciences (SLU) Uppsala, Sweden
